# Obesity is Associated With Poor Surgical Outcome in Crohn’s Disease

**DOI:** 10.4021/gr553w

**Published:** 2013-07-14

**Authors:** Talha A. Malik, Ashish Manne, Robert A. Oster, Austin Eckhoff, Seidu Inusah, Alexandra M. Gutierrez

**Affiliations:** a Division of Gastroenterology/Hepatology, Department of Medicine, University of Alabama at Birmingham, USA; b Division of Preventive Medicine, Department of Medicine, University of Alabama at Birmingham, USA; c Department of Biostatistics, University of Alabama at Birmingham, USA; d Division of Gastroenterology/Hepatology, Department of Medicine, Washington University in Saint Louis, USA

**Keywords:** Obesity, Body mass index, Crohn’s disease, Surgery

## Abstract

**Background:**

Published data suggest a link between obesity and adverse outcomes in Crohn’s disease (CD). We aimed to test the hypothesis that obese CD patients would be more likely than non-obese CD patients to have poor surgical outcome when undergoing surgery for a complication of CD.

**Methods:**

We designed a retrospective cohort study to test our hypothesis. The population comprised of adult CD patients who underwent CD related surgery at a tertiary referral center. The exposed and unexposed cohorts were represented by patients who were obese vs. non-obese at the pre-op visit respectively. Outcome was represented by successful vs. unsuccessful surgical outcome as deemed by the treating clinician.

**Results:**

Ninety CD patients were eligible for inclusion into this cohort study of which 36 were obese (exposed cohort) and 54 were non-obese (unexposed cohort). Among obese CD patients, 64% had an unsuccessful surgical outcome vs. 41% with unsuccessful surgical outcome among the non-obese. Based on unadjusted bivariate analysis, potential confounders identified included age and type of surgery. Gender distribution, disease duration, ethnicity, tobacco use, steroid use, traditional and biological immune modulator use and clinical disease activity were similar between the two groups. Logistic regression adjusted for age and type of surgery revealed that obese CD patients were approximately 2.5 times more likely to have a poor surgical outcome than patients with CD who were not obese (P = 0.05 OR 2.53 95% CI 0.99 - 6.52). BMI as a continuous variable (adjusted for age and type of surgery) appeared to be associated with poor surgical outcome (P = 0.06 OR 1.07 95% CI 0.99 - 1.15).

**Conclusions:**

Obesity may be associated with poor surgical outcome in CD patients.

## Introduction

Crohn’s Disease (CD) is a chronic inflammatory condition, primarily of the gastrointestinal system that affects 750,000 people in the US. It is characterized by transmural inflammation and can involve any part of the intestinal tract [1, 2]. Its presentation is heterogeneous in that its clinical course is variably characterized by intestinal inflammation, stricturing of segments of the bowel causing obstruction and/or occurrence of penetrating mucosal complications leading to intestinal perforation and formation of fistulas and/or abscesses [3-5]. While medical management is the cornerstone of therapy in almost all forms of CD, surgery is an inevitable treatment modality as up to 80% of CD patients undergo operative intervention during the course of their disease [3-5].

Adipose tissue is an active endocrine organ that is made up of elements of connective tissue as well as of cells represented by pre-adipocytes and adipocytes that may be prominent mediators of inflammation in the human body [6-9].Abnormally increased stores of adipose tissue in the body represent obesity which is measured as increased body weight to height ratio, also referred to as the body mass index (BMI). Standard weight is defined as BMI of 18.5 - 24.9 kg/m^2^. BMI of 25.0 - 29.9 kg/m^2^ has traditionally represented overweight or pre-obesity. Obesity is defined as BMI of greater than 30 kg/m^2^ [6].

The importance of the need to study the association between the obese state and CD outcomes emanates from the observation that there is credible molecular evidence for a link between increased mesenteric adipose stores and intestinal inflammation in CD [8, 9]. Moreover, in the past few decades, obesity has undergone a marked increase in prevalence within the US and across the globe, and this increased trend of obesity is also reflected in patients with CD [10-12].

Whether the molecular association between increased mesenteric adipose tissue and intestinal inflammation in CD truly translates into a relationship between the two entities in clinical practice is either not well known yet or at best, research on it is equivocal. There has been some clinical research evaluating an association between increased adipose tissue represented clinically by the obese state and several outcome parameters in CD such as clinical and mucosal disease activity as well as surgical outcomes, but further work needs to be done before these research questions can be adequately answered [13-15].

The objective of this retrospective cohort study was to offer a unique perspective by systematically evaluating the effect of pre-op BMI on post-op outcome in patients with CD.

## Methods

### Study design

This was a retrospective cohort study.

### Patients

Men and women 19 years and older with CD who underwent surgery for a complication related to CD at University of Alabama at Birmingham Hospital (UAB) between 2000 and 2009 were retrospectively identified using a health system billing search engine. In order to assure higher sensitivity, surgical log books were reviewed to identify additional patients.

Patients screened based on search criteria had to meet further inclusion and exclusion criteria. Eligible patients had to have established endoscopic or histological diagnosis of CD in addition to having surgery for a well-categorized CD related complication between 2000 and 2009. Data on pre-operative BMI, demographics and post-surgical outcome in the patients had to be available for them to be included into the study. Patients were excluded if they had a BMI of less than 18.5 kg/m^2^, if they had any current history of cancer, were receiving chemotherapy or had missing data.

### Data collection

Information obtained from review of patient charts included: 1), demographic data (age, ethnicity, gender, and the body mass index); 2), clinical variables (age, smoking history, duration of disease and clinical disease activity at the time of surgery); 3), medication history (use of steroids, traditional immune modulators and biological agents); 4), type of surgery and 5), surgical outcome.

Data were collected from medical records onto a standard data collection form and then entered into a database. All recorded data were reviewed twice to ensure accuracy.

### Definitions of variables

#### Body mass index

Obesity was measured using body mass index (BMI). BMI was calculated by dividing a person’s weight in kg by height in meters squared. Based on the BMI, patients were divided into two groups. Obese patients were those with a BMI of greater than 30 kg/m^2^. The rest had a BMI between 18.5 - 29.9 kg/m^2^ and they were classified as non-obese. Pre-op height and weight were used to calculate BMI. Self- reported height and weight were not used to avoid recall bias.

#### Type of surgery

Surgery was categorized into one of three types according to invasiveness of each kind of intervention. The first category comprised of Seton placement (with or without a preceding fistulotomy), or incision and drainage for an associated perianal abscess. The second type of surgery was classified as an advancement flap, whether it was mucosal or submucosal, and with or without an associated circular or a gracilis flap. Internal strictureplasty, internal abscess drainage and internal balloon dilations were also considered under the second type of surgery. The third category comprised of resective surgeries and included ileocecectomy, ileocolonic resection, colectomy and proctocolectomy. Fecal diversion procedures (ileostomy/colostomy) and abdominal perineal resections were also classified under the third type of surgery. Patients who underwent two categories of procedures were classified under the more invasive category.

#### Clinical disease activity

Crohn’s disease clinical activity was documented for each patient in accordance with American College of Gastroenterology practice guidelines. Patients were divided into two categories, those in remission or mild to moderate disease, and those with moderate to severe disease [4].

#### Medication history

History of corticosteroid use was defined as more than one week of conventional oral or parenteral steroids, or more than a month of rectal or topical steroids or more than 3 months of oral budesonide within the past one year. Any history of oral, parenteral, rectal, topical steroids including use of oral budesonide within the one month prior to surgery was considered recent steroid use. Immune modulator use was considered positive if a patient was on azathioprine (AZA), 6-mercaptopurine (6-MP), methotrexate (MTX) or any biological agent prior to or at the time of surgery and was on the particular agent at the first post-op follow-up appointment.

#### Tobacco use

Smokers were defined as those who were smoking at time of surgery.

### Defining outcome

Outcome of interest was unsuccessful surgical outcome at or before the one month post-op visit as defined by the treating or managing clinician. Specifically, unsuccessful or poor surgical outcome was defined as any post-op complication elicited during the post-op recovery period, or during the first post-op scheduled or unscheduled visit that required a non-standard intervention. Minor complications were characterized by post-op wound infections, delayed wound healing or prolonged recovery. Major complications were defined as anastomotic leak, wound dehiscence, development of an intra-abdominal abscess or death. Any patient who did not develop any of these complications was considered to have had a successful surgical outcome.

### Statistical analysis

#### Descriptive analysis

Descriptive data on demographic and clinical information including data on exposure and outcome variables were collected for the entire cohort.

#### Primary bivariate analysis

Bivariate analysis comparing exposure of interest with its co-variates and with the outcome of interest was completed in order to select the co-variates to be placed in the adjusted regression model.

#### Multivariable analysis

Multivariable Logistic Regression Analysis was used to calculate the adjusted odds ratio for the odds of unsuccessful surgical outcome vs. successful surgical outcome based on the exposure of interest, obesity.

#### Statistical analysis software

Statistical software (SAS, version 9.2; SAS Institute, Inc., Cary, NC) was used to perform all statistical analyses. Statistical tests were two-sided with a 5% significance level (namely, α = 0.05).

### Study approval

This study was approved by University of Alabama’s Institutional Review Board’s Institutional Review Board.

## Results

A total of 90 patients were eligible for inclusion into this retrospective cohort study. Comparison of demographic and clinical characteristics among the 36 exposed (obese) vs. 54 unexposed (non-obese) patients appears in Table 1. In the entire cohort, 45 (50%) CD patients had poor surgical outcome based on review by treating clinician at or before the one month post-op visit with 64% of them being obese vs. 41% who were non-obese.

**Table 1:** Comparison of Obese and Non-obese Patients (Also Appears Within Text)

	Obese N = 36 (%)	Non-obese N = 54 (%)	P-value
BMI at surgery- median (range)	32.3 (30.1 - 72.4)	23.3 (18.5-28.5)	< 0.0001*
Age at surgery -median (range)	45 (19 - 88)	34 (19 - 75)	0.001*
Disease duration-median (range)	8 (1 - 41)	9 (1 - 36)	0.644
Type of surgery			0.079∧
Fistulotomy, Seton Placment	5 (14%)	19 (35%)	0.025*
Flap, Strictureplasty, Dilation	6 (17%)	6 (11%)	0.448
Resection, Diversion	25 (69%)	29 (53%)	0.135
Gender (male)	9 (25%)	21 (39%)	0.171
Ethnicity			0.146
Caucasian	23 (64%)	41 (76%)	0.217
African American	9 (25%)	12 (22%)	0.76
Tobacco Use	9 (25%)	13 (24%)	0.92
Moderate to Severe Disease	17 (47%)	19 (35%)	0.254
Steroid use	10 (28%)	19 (35%)	0.461
Immune modulator use	28 (78%)	48 (89%)	0.154
Azathioprine/6-MP	13 (36%)	21 (39%)	0.79
TNF blocker	17 (47%)	25 (46%)	0.931
Methotrexate	1 (3%)	8 (15%)	0.08∧

*P-value < 0.05; ∧P-value < 0.10.

Based on bivariate analysis looking at clinically relevant co-variates, potential confounders for the association between obesity and surgical outcome identified included age and type of surgery (P < 0.1). Gender distribution, disease duration, ethnicity, tobacco use, steroid use, traditional and biological immune modulator use and clinical disease activity were similar between the exposed (obese) and unexposed (non-obese) cohort and therefore were not included into the adjusted model (Table 1). Unadjusted logistic regression analysis examining the association between obesity and surgical outcome in CD demonstrated a statistical significant relationship (P = 0.03 OR 2.57 95% CI 1.08 - 6.14). Unadjusted logistic regression analysis that assessed the association between BMI as a continuous variable and surgical outcome in CD also demonstrated results that displayed a trend towards significance (P = 0.07 OR 1.07 95% CI 0.99 - 1.15).

**Figure 1:**
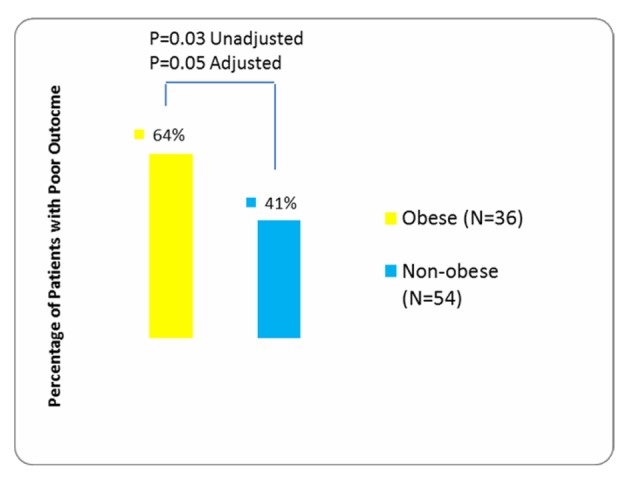
Comparison of outcome in obese vs. non-obese patients (also appears within text).

Multivariable stepwise logistic regression analysis adjusted for age and type of surgery revealed that obese CD patients were approximately 2.5 times more likely to have a poor surgical outcome than patients with CD who were not obese (P = 0.05 OR 2.53 95% CI 0.99 - 6.52) (Figure 1). BMI as a continuous variable (adjusted for age and type of surgery) also predisposed CD patients to a poor surgical outcome (P = 0.06 OR 1.07 95% CI 0.99 - 1.15), with every unit increase in BMI associated with a 7% higher probability of having an unsuccessful surgical outcome.

## Discussion

The most significant finding observed in this retrospective cohort study was that obese CD patients appeared to be more likely to have a poor surgical outcome when compared to CD patients who were not obese. It was also suggested as a result of marginal statistical significance that an increase in BMI itself may also predict a poor surgical outcome in patients with CD.

Review of past literature reveals that there is a paucity of knowledge on the influence of increased BMI on the clinical presentation and course of CD patients. A 2002 French study by Blain and colleagues compared the clinical course of CD patients who were obese with those CD patients who were not obese [13]. They defined obese CD patients as those who had a BMI of greater than 25 kg/m^2^ at the time of disease onset and more than 30 kg/m^2^ at any time during the course of their disease thereafter. They observed that obese CD patients had a higher rate of disease flares and hospitalizations. But they did not detect any difference between obese and non-obese CD patients based on long-term outcome which included outcome after resective surgery in those patients who underwent surgery [13]. Trend of hospitalizations or flares were not looked at in our study. We did detect a non-significant increased rate of clinical disease activity in obese patients (47%) compared to patients who were not obese (35%) (P = 0.254).

A 2006 US study by Hass et al also evaluated the role of BMI in predicting the clinical course of CD patients [14]. They compared overweight or obese CD patients, namely those who had a BMI of greater than 25 kg/m^2^ to CD patients who had a normal or a low BMI of less than 25 kg/m^2^. Within the group of patients with BMI < 25 kg/m^2^, the subset representing patients who had a BMI of less than 18.5 kg/m^2^ were also included. In their study, the degree of therapy escalation required did not differ in the two CD groups as opposed to the study by Blain and colleagues in which they had observed a greater likelihood of flares and hospitalization in CD patients who were obese. Of note, Blain et al had compared obese patients to those who were non-obese whereas Hass and colleagues had combined overweight and obese patients into one group. Interestingly, Hass et al did find that when compared to the subset of patients who had a BMI of less than 18.5 kg/m^2^, overweight/obese patients (BMI of greater than 25 kg/m^2^) had a much shorter time to first surgical intervention [14]. Underweight CD patients were not included in the studied patient population because during the initial pre-screening, there were only three patients who had a BMI of less than 18.5 kg/m^2^ and thus they were excluded.

On comparing the sub-groups of standard weight (BMI of 18.5 - 24.9 kg/m^2^) and overweight (BMI of 25 - 29.9 kg/m^2^) patients within our non-obese population, it was observed that the difference in surgical outcome between them was not significantly different. The slightly improved, albeit non-significant surgical outcome in the overweight population compared to those who were of standard weight may be because the current criteria for dividing individuals between normal weight and overweight are perhaps too stringent. The most ill CD patients characteristically suffer from undernourishment and loss of weight. When CD patients are in remission or have only mild symptoms, they gain weight. It is entirely possible that during their healthiest period, they cross the barrier between standard weight and overweight. Moreover, BMI is not able to reliably distinguish between muscle weight and weight from fat. It is therefore also entirely possible that many overweight CD patients in our patient population represented the healthiest patients in our cohort. Lastly, BMI may not be a reliable surrogate for mesenteric fat content.

Retrospective design, lack of a group representing underweight CD patients and a relatively small sample size were among several important limitations of our study. Also, the lack of homogeneity in regard to type of surgery was another limitation that was partially accounted for by inclusion of this clinically important factor into the adjusted multivariable regression model. Confounding by indication was another concern in this study. For example, more severe cases may have been more likely to be on biological immune modulators. At the same time, they may also have had better disease control at the time of surgery. This element was probably not a major confounder in our study as the rates of traditional and biological immune modulators were similar in the obese and non-obese patients. Moreover, data on previous surgeries and number of previous surgeries were also not included in our analysis; this information may have impacted outcomes in individual CD patients.

In order to make further progress on this topic, there is a need for larger studies, preferably prospective that include a larger spectrum of BMI groups as well as a larger patient population in each respective group. Additionally, larger studies will allow inclusion of more co-variates into adjusted models to better account for potential confounding and will have additional statistical power to detect important differences between groups.

Finally, in order to better identify the threshold of increased post-operative risk in regard to BMI, future studies should also consider comparing the clinical course and outcome of obese CD patients on one side to the clinical course and outcome of overweight and normal weight CD patients combined as one group on the other.

## Abbreviations

CD: Crohn’s disease
BMI: Body mass index
TNF-alpha: Tumor necrosis factor alpha
AZA: Azathioprine
6-MP: 6-mercaptopurine
MTX: Methotrexate
SAS: Statistical analysis software
